# Supplementary material for: Enhancing coevolutionary signals in protein–protein interaction prediction through clade-wise alignment integration
Source: Sci Rep. 2024 Mar 12;14:6009. doi: 10.1038/s41598-024-55655-9 (PMC10933411; doi:10.1038/s41598-024-55655-9)
Supplement: Supplementary file 1 — Supplementary Figures. [file 41598_2024_55655_MOESM1_ESM.docx]

Enhancing coevolutionary signals in protein-protein interaction prediction through clade-wise alignment integration

Tao Fang^1,2^, [Damian Szklarczyk](https://bmcbioinformatics.biomedcentral.com/articles/10.1186/s12859-019-2828-z#auth-Damian-Szklarczyk)^1,2^, Radja Hachilif^1,2^, [Christian von Mering](https://bmcbioinformatics.biomedcentral.com/articles/10.1186/s12859-019-2828-z#auth-Christian_von-Mering)^1,2^*

^1^Department of Molecular Life Sciences, University of Zurich, 8057 Zurich, Switzerland

^2^SIB Swiss Institute of Bioinformatics, 1015 Lausanne, Switzerland

*corresponding author. email: [mering@mls.uzh.ch](mailto:mering@mls.uzh.ch);

# Supplementary Information

Table S1: Predicted direct PPs with high interaction signals according to our RF model as well as AlphaFold-Multimer.

**Supplementary Fig. 1 Negative control: shuffling MSA half-rows destroys the coevolution signal.**

**
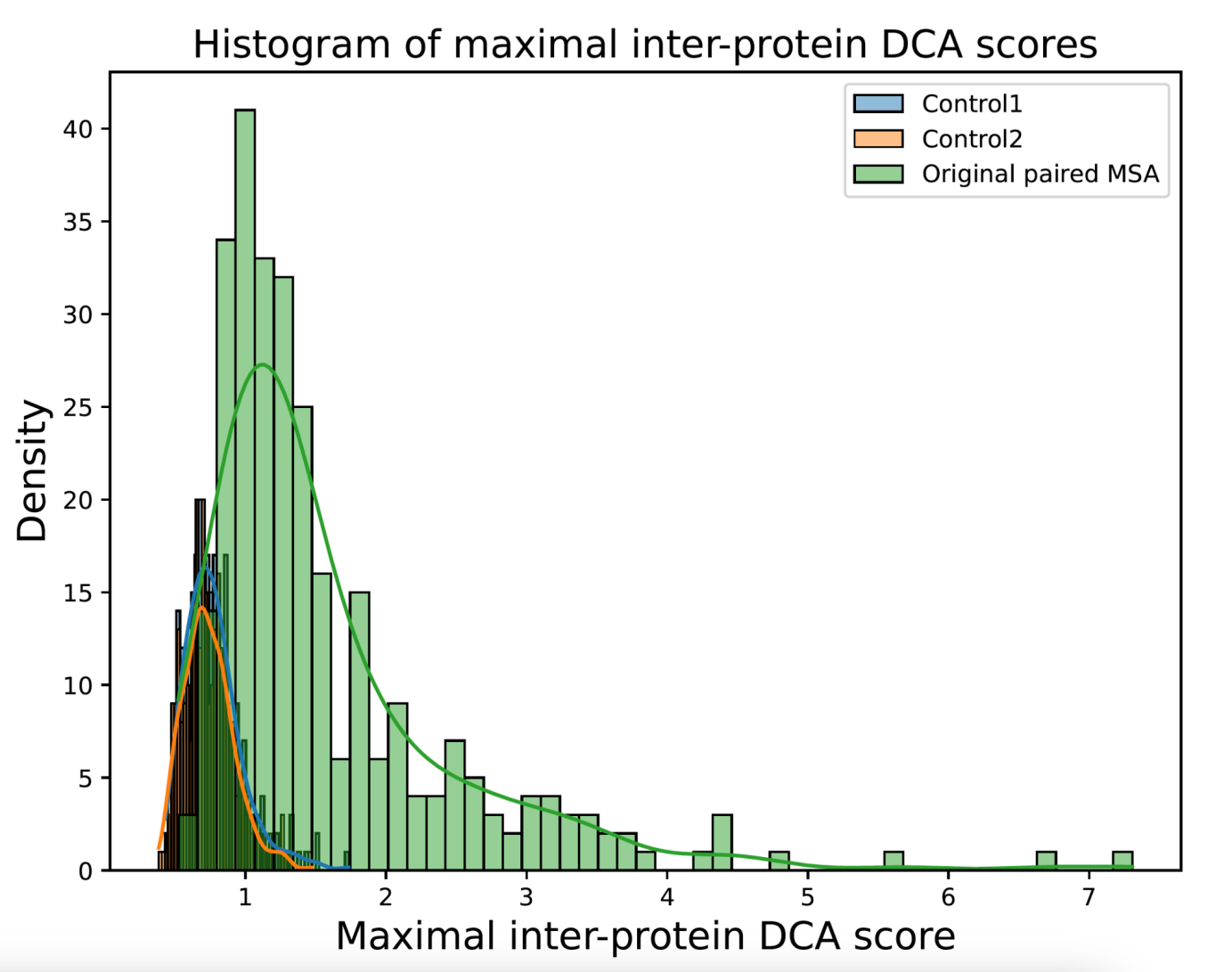
**

This histogram displays the distribution of maximal inter-protein DCA scores from a DCA analysis on a curated PPI benchmark derived from PDB complexes. 'Original paired MSA' denotes MSAs constructed at the same phylum level, with potentially interacting interologs aligned in corresponding rows. 'Control 1' refers to MSAs where the first protein's MSA row positions are unchanged, but the second protein's MSA row positions are randomly shuffled. 'Control 2' indicates MSAs where the first protein's MSA remains constant, while the second protein's MSA, comprising all available bacterial sequences, is randomly shuffled and sampled for pairing. As can be observed, randomly pairing proteins from different species reduces the DCA-inferred inter-protein coevolution signals substantially.

**Supplementary Fig. 2 Sequence space (pairwise sequence identity distributions) after MSA downsampling, and within different phyla.**


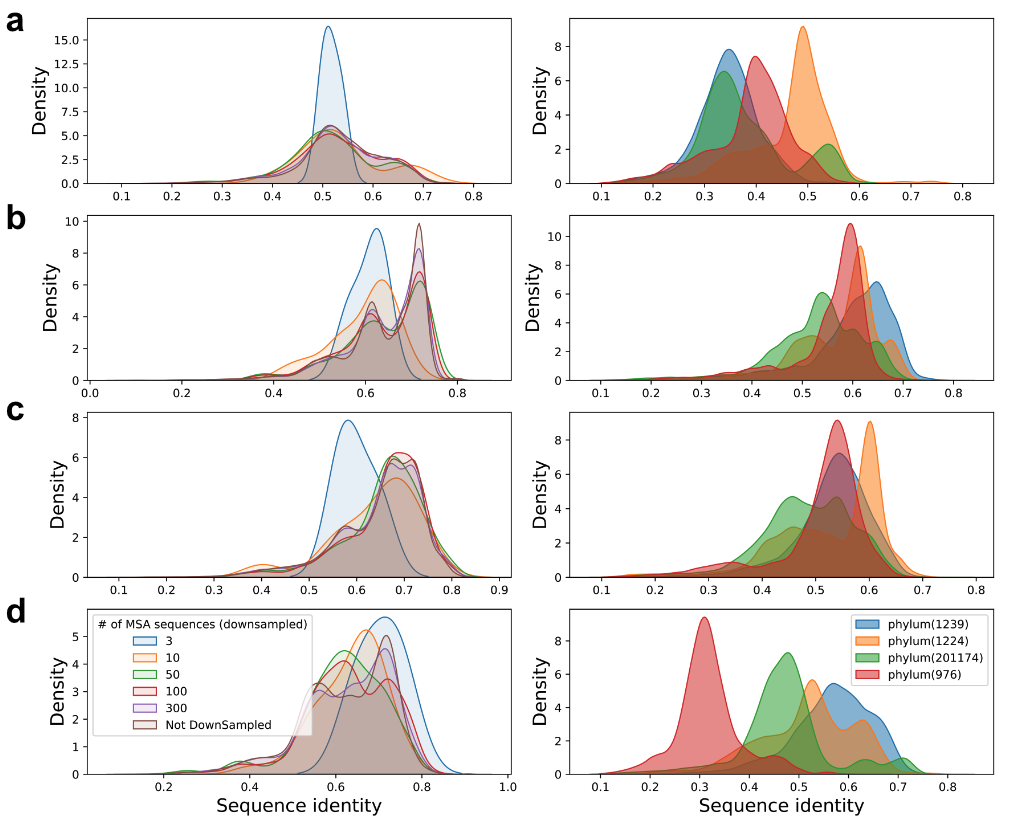


Distribution plots of pairwise sequence identities in paired MSAs with different extents of downsampling (left panels) and in different phyla (right panels). Here, we arbitrarily chose four different protein-protein interaction types as representatives: **a** small ribosomal subunit protein rpsB and rpsE. **b** Protein translocase subunit SecY and Membrane protein insertase YidC. **c** Transcription antitermination protein NusB and Transcription termination / antitermination protein NusA. **d** Malonyl CoA-acyl carrier protein transacylase fabD and Acyl carrier protein acpP. The distribution of pairwise sequence identities in paired MSAs indeed varies widely at different clades of the tree of life, illustrated by different phyla at the right panel. Conversely, random downsampling tends to keep the pairwise sequence identity distributions stable, illustrated by the left panel (with the exception of downsampling to size 3, resulting in more shifts due to the stochasticity of the sampling).

**Supplementary Fig. 3 Separating out the independent phylogenetic cooccurrence signal.**


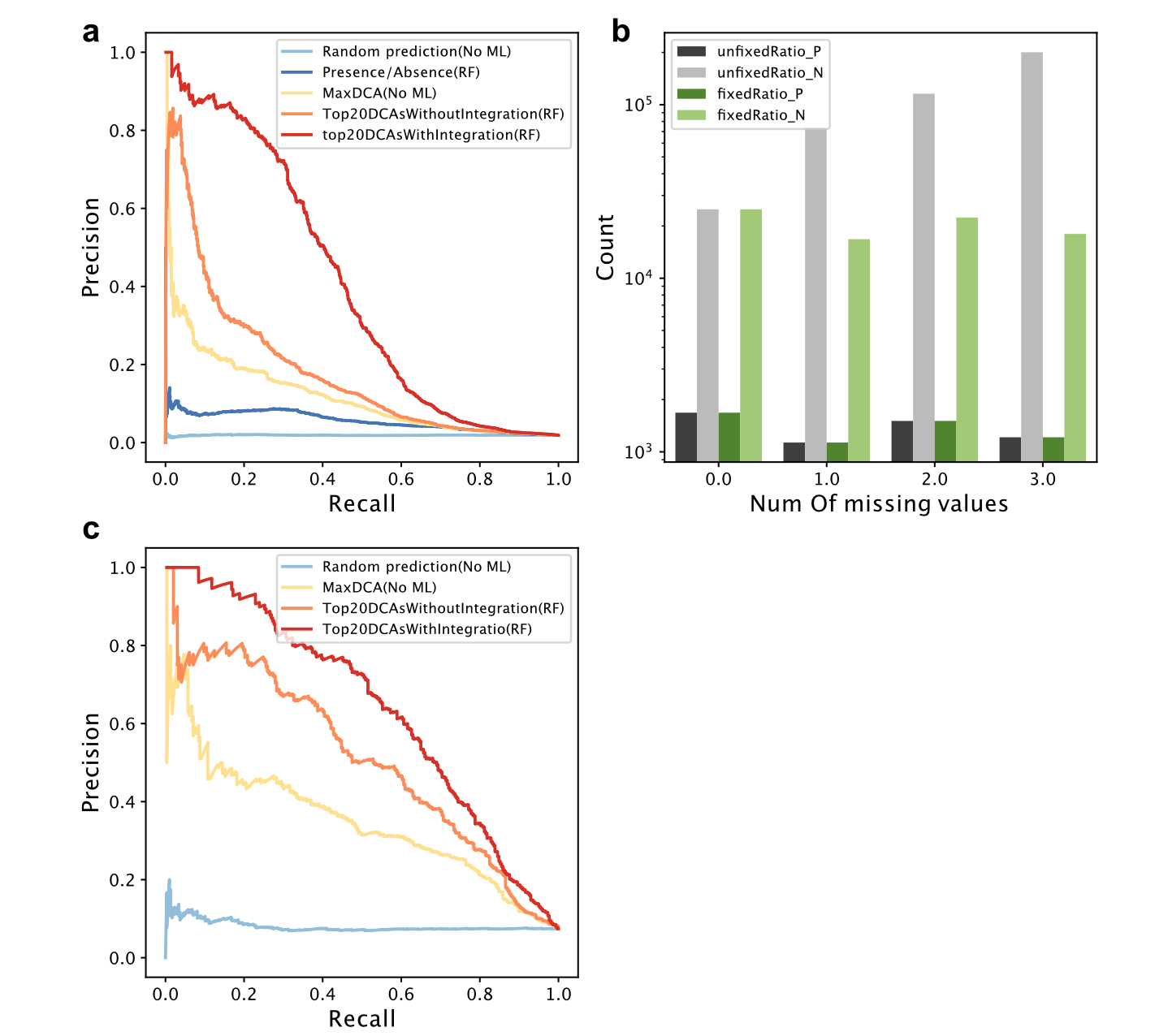


**a** PR (Precision-Recall) curve of coevolution signal integration prediction performance on the initial benchmark. **b** Number of negative and positive samples on the benchmarks for coevolution signal integration before and after fixing the ratio of the number of negative and positive samples in different sample groups according to their missing values. **c** PR curve of prediction performance of coevolution signal integration on the benchmark after removing all samples with any missing orthologous protein pairs in subject species.

**Supplementary Fig. 4 Prediction performance of coevolution signal integration at proteome level after removing training samples proportionally**.


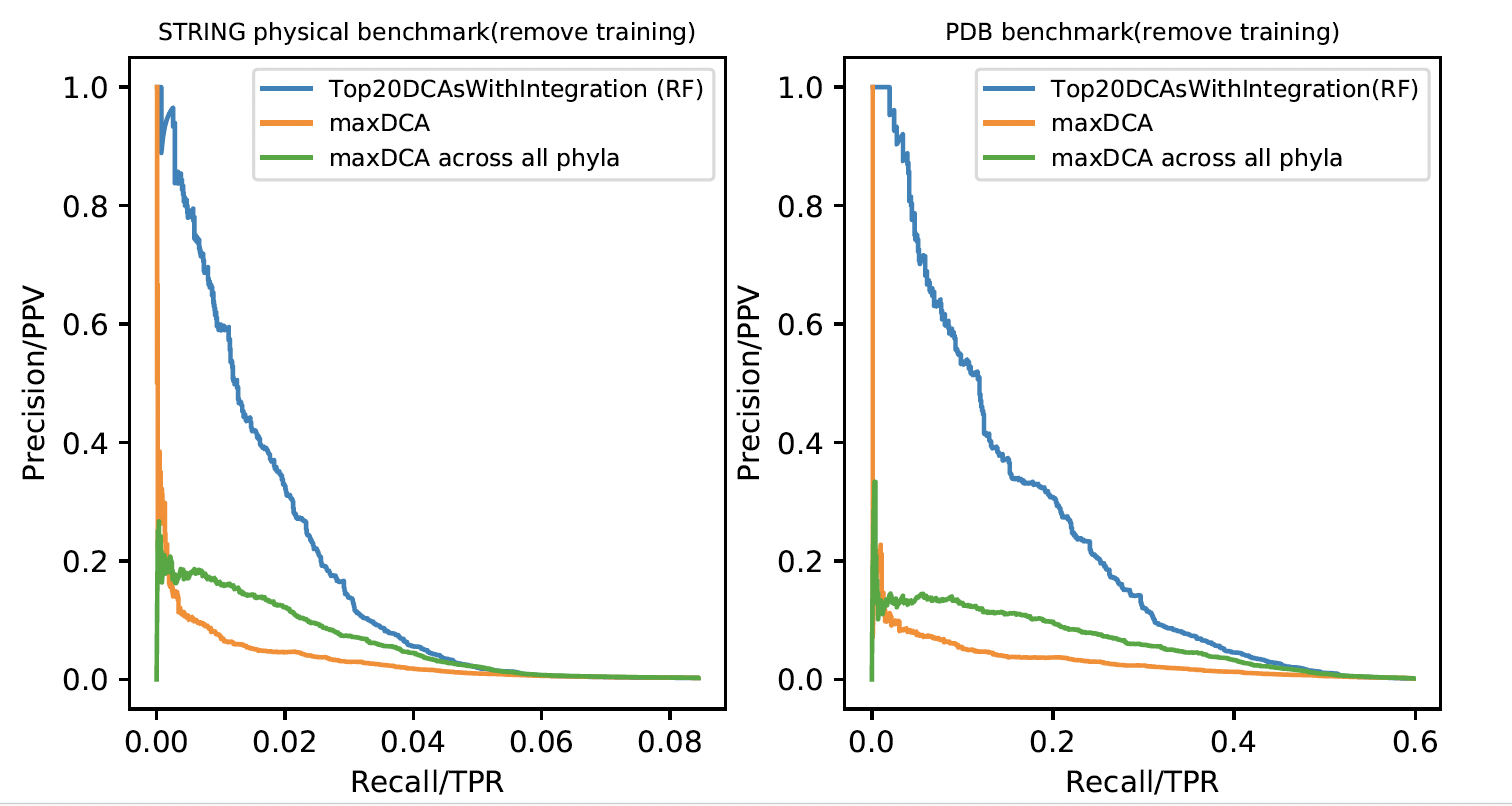


**Supplementary Fig. 5 Comparison between coevolution and AlphaFold performance.**


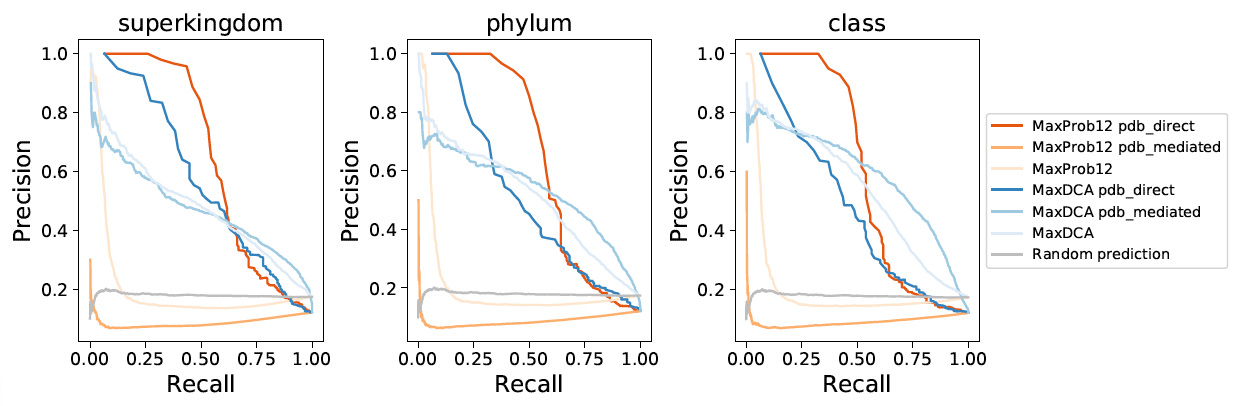


Precision-Recall curve of prediction performance of max coevolution signal and maximal AlphaFold contact probability on the common pdb_direct and pdb_mediated benchmarks, at different taxonomic levels.
